# Comparative Analysis of Biochemical and Lipid Profile Markers at Different Stages of Lung Cancer: A Cross-Sectional Study

**DOI:** 10.7759/cureus.75737

**Published:** 2024-12-15

**Authors:** Rajyalakshmi Gogineni, Suresh Arumugam, Natrajan Muninathan, Kuppusamy Baskaran

**Affiliations:** 1 Biochemistry, Meenakshi Medical College Hospital and Research Institute, Meenakshi Academy of Higher Education and Research, Kanchipuram, IND; 2 Microbiology, Meenakshi Medical College Hospital and Research Institute, Meenakshi Academy of Higher Education and Research, Kanchipuram, IND

**Keywords:** biomarkers, crp, ecog performance status, il-6, lipid profile, lung cancer, pth, systemic inflammation

## Abstract

Background: Systemic inflammation, metabolic dysregulation, and changes in biochemical markers are closely associated with the progression of lung cancer. This study focuses on evaluating serum parathyroid hormone (PTH), C-reactive protein (CRP), lipid profile parameters, and interleukin-6 (IL-6) in relation to the stages of lung cancer, exploring their potential as biomarkers for assessing disease severity.

Methods: A total of 160 lung cancer patients were selected for a cross-sectional study and equally distributed into four clinical stages (Stages 1-4). PTH, CRP, low-density lipoproteins (LDLs), high-density lipoproteins (HDLs), triacylglycerides (TAGs), and IL-6 were analyzed in serum by the enzyme-linked immunosorbent assay, chemiluminescent immunoassay, and enzymatic techniques. Statistical analysis included analysis of variance with post hoc Tukey’s test and Pearson correlation analysis for body mass index (BMI) and Eastern Cooperative Oncology Group (ECOG) performance status.

Results: PTH levels increased significantly as the disease progressed (Stage 1: 10.8 ± 2.5 pg/mL; Stage 4: 498 ± 89.4 pg/mL; p < 0.001). Similarly, CRP (Stage 1: 5.8 ± 1.2 mg/L; Stage 4: 8.6 ± 2.4 mg/L; p = 0.004) and IL-6 (Stage 1: 3.8 ± 0.9 pg/mL; Stage 4: 8.5 ± 2.1 pg/mL; p < 0.001) levels showed significant increases, indicating elevated systemic inflammation in the advanced stages. Lipid profile analysis revealed a decrease in HDL levels (p = 0.042) and an increase in TAG levels (p = 0.031), while LDL levels did not show statistically significant variations (p = 0.085). The ECOG performance status significantly deteriorated with advancing stages (p = 0.001) and was strongly correlated with BMI (r = 0.915; p < 0.001).

Conclusion: Lung cancer progression is strongly linked to elevated levels of PTH, CRP, and IL-6, alongside dysregulated lipid profiles. The interplay of systemic inflammation and metabolic alterations underscores key pathophysiological mechanisms underlying the disease. Emerging evidence suggests that PTH and IL-6 hold promise as potential biomarkers for assessing disease severity and progression.

## Introduction

Despite being one of the leading causes of cancer morbidity and mortality worldwide, lung cancer is still diagnosed in a late stage and has a poor prognosis [1]. Lung cancer is classified broadly into small cell lung cancer and non-small cell lung cancer (NSCLC), with NSCLC being the most prevalent subtype of the disease [2]. Despite the progress made in diagnostic and therapeutic methods, the survival rates for lung cancer remain poor, particularly in the late stages. Thus, it is crucial to identify reliable biomarkers for early detection, prognosis, and monitoring of disease progression [3]. Increasingly, systemic inflammation and metabolic dysregulation have been recognized to be involved in cancer progression [4].

Tumor-related inflammation is indicated by elevated levels of inflammatory markers (e.g., C-reactive protein, CRP; interleukin 6, IL-6) and has been linked to poor outcomes in a number of malignancies, including lung cancer [5]. Parathyroid hormone (PTH) has emerged as a promising biomarker because of its involvement in calcium metabolism and cancer progression [6]. Another hallmark of cancer is alteration in lipid metabolism, including changes in lipid profiles, including LDL, HDL, and triacylglycerides (TAGs), in different malignancies [7]. These metabolic changes likely represent an increased energy demand by the tumor as well as systemic metabolic disturbances and highlight their potential as diagnostic and prognostic tools [8].

The primary objective of this study was to evaluate and compare the serum levels of PTH, CRP, lipid profile parameters, and IL-6 across different stages of lung cancer. This approach aims to elucidate the dynamic changes in these biochemical markers with disease progression based on current evidence, highlighting their roles in systemic inflammation, metabolic dysregulation, and tumor-associated pathophysiology. By correlating these markers with disease stages, the study seeks to contribute to a better understanding of their potential utility in early diagnosis, prognosis, and monitoring of lung cancer progression. We investigated these biomarkers to understand how they relate to disease progression and their use in clinical practice. The study also investigated the association between ECOG performance status, a functional impairment measure, and body mass index (BMI) to discover additional clinical features of lung cancer patients.

## Materials and methods

Study design

This cross-sectional study was carried out at the Tammannagari Ramkrishna Reddy (TRR) Institute of Medical Sciences, Hyderabad, and the Rajaboina venkataiah Memorial (RVM) Institute of Medical Sciences and Research Center, Siddipet, over a 30-month period (March 2022 to August 2024). The sample size for this study was determined based on statistical calculations to ensure sufficient power for detecting differences in serum biomarker levels across lung cancer stages. Using a one-way analysis of variance (ANOVA) to compare means, the required sample size was calculated to detect a medium effect size (f = 0.25) with 80% power and a significance level (α) of 0.05. This calculation indicated a minimum of 36 participants per group. To account for potential dropouts or incomplete data, the sample size was increased to 40 patients per stage, resulting in a total of 160 participants. Patients were recruited consecutively from the outpatient and inpatient departments of the TRR Institute of Medical Sciences, Hyderabad, and the RVM Institute of Medical Sciences and Research Center, Siddipet, during the study period. Equal allocation across the four stages of lung cancer (Stages 1-4) was achieved by enrolling patients sequentially as they presented, ensuring a balanced representation for statistical comparisons. Serum levels of PTH, CRP, and lipid profile parameters like low-density lipoprotein (LDL), high-density lipoprotein (HDL), TAG, and IL-6 were measured using standard laboratory techniques, including enzyme-linked immunosorbent assay (ELISA) and chemiluminescent immunoassay (CLIA). Demographic and clinical data were collected, and associations between these biomarkers, BMI, and ECOG performance status were analyzed. 

Inclusion Criteria

The study included patients diagnosed with lung cancer who were actively undergoing treatment at the designated study sites. Only those patients who provided informed consent to participate were considered eligible for inclusion.

Exclusion Criteria

Patients with cancers other than lung cancer were excluded from the study. Additionally, individuals with severe comorbid conditions that could interfere with the study parameters and those who did not provide informed consent were also excluded.

Sample collection and biochemical analysis

Venous blood samples were collected from all participants using standard venipuncture techniques to ensure consistency and reduce procedural variability [9]. Immediately after collection, the blood samples were centrifugated at 3,000 rpm for 15 minutes to separate the serum from cellular components [9]. The extracted serum was then aliquoted and stored at -80°C to maintain the stability of the analytes until analysis.

For the measurement of PTH levels, a sensitive CLIA was employed due to its high specificity and ability to detect low concentrations of hormones in the serum [10]. IL-6, a proinflammatory cytokine implicated in cancer progression, was quantified using a standardized ELISA, following the manufacturer's protocols to ensure accuracy and reproducibility [10].

CRP levels were determined using a high-sensitivity immunoassay, which can detect minute changes in CRP concentrations, thereby providing insights into low-grade systemic inflammation associated with malignancies [11]. This method is widely recognized for its precision in clinical and research settings.

The lipid profile, including measurements of LDL, HDL, and TAG, was assessed using automated enzymatic methods on a fully automated biochemistry analyzer [12]. These assays are considered the gold standard for lipid profiling due to their accuracy, efficiency, and reproducibility in detecting lipid abnormalities that may correlate with cancer progression.

Statistical analysis

Data were analyzed using IBM SPSS Statistics, version 25.0 (IBM Corp., Armonk, NY). The mean and standard deviation of all parameters were calculated for each stage of lung cancer to summarize the data distribution. One-way ANOVA was employed to assess differences in means across the four stages, as it is a robust statistical method for comparing means among multiple groups. This was followed by post hoc Tukey’s test to identify specific pairs of groups with significant differences, ensuring accurate pairwise comparisons while controlling for type I error. Pearson's correlation coefficient was calculated to evaluate the relationship between BMI and ECOG performance status, as it is a widely used measure for assessing the strength and direction of linear associations between continuous variables. A p value of <0.05 was considered statistically significant, aligning with conventional thresholds in biomedical research.

Ethical considerations

The study protocol was approved by the Institutional Ethics Committee of the TRR Institute of Medical Sciences (TRRIMS/Research/015/2022; dated 5/1/2022). Written informed consent was obtained from all participants. Confidentiality and anonymity were maintained throughout the study. Patient confidentiality and data protection were ensured by adhering to strict ethical standards. Written informed consent was obtained from all participants after they were provided with comprehensive information about the study's objectives, procedures, and rights. To maintain anonymity, all patient data were deidentified using unique codes, and no personally identifiable information was included in the analysis or reporting. Access to the data was restricted to authorized research personnel, and all data were securely stored in password-protected systems. The study protocol was reviewed and approved by the Institutional Ethics Committee, ensuring compliance with ethical guidelines for confidentiality and data security.

## Results

Demographic and clinical characteristics

The mean age showed a significant increase with disease progression, from 57 ± 8.2 years in Stage 1 to 65 ± 11.5 years in Stage 4 (p = 0.012). ECOG performance status also demonstrated statistically significant differences across stages (p = 0.001), with poorer functional performance (≥2) being more prevalent in Stage 4. While BMI did not vary significantly across stages (p = 0.337), it exhibited a strong positive correlation with ECOG performance status (r = 0.915, p < 0.001), indicating that patients with higher BMI tended to maintain better functionality regardless of disease stage.

The analysis of categorical variables, including gender, smoking status, and histological type, highlighted stage-specific trends, with 95% confidence intervals (CIs) enhancing the precision of these findings. BMI CIs narrowed progressively across stages, while age CIs widened, reflecting greater variability in older patients as the disease advanced. For ECOG performance status, the CIs for scores of 0-1 decreased from 66.24%-87.76% in Stage 1 to 27.04%-47.96% in Stage 4, while the CIs for scores of ≥2 increased from 12.24%-33.76% in Stage 1 to 52.04%-72.96% in Stage 4. These trends, confirmed through chi-square and ANOVA tests, underscore the importance of ECOG as a robust indicator of disease progression and functional decline (Table 1).

**Table 1:** Demographic and clinical characteristics of the recruited lung cancer patients F values for the comparisons were estimated based on the respective p values. For age, the F value ranged between 3.5 and 5.0. For BMI, the F value was <1, indicating no significant difference. For ECOG performance status, the F value ranged between 6.0 and 9.0. A strong positive correlation (r = 0.915) was observed between BMI and ECOG performance status SD: standard deviation; COPD: chronic obstructive pulmonary disease; BMI: body mass index; ECOG: Eastern Cooperative Oncology Group

Characteristics	Total, n (%), 160 (100%)	Stage 1, n (%), 40 (25%)	Stage 2, n (%), 40 (25%)	Stage 3, n (%), 40 (25%)	Stage 4, n (%), 40 (25%)	p value
Age (years)						
Mean ± SD	61 ± 9.5	57 ± 8.2	60 ± 9.1	63 ± 10.2	65 ± 11.5	0.012
Range	42-81	41-70	45-75	46-78	50-82	
Gender						
Male	112 (70%)	27 (67.5%)	28 (70%)	30 (75%)	27 (67.5%)	0.921
Female	48 (30%)	13 (32.5%)	12 (30%)	10 (25%)	13 (32.5%)	
Smoking status						
Current smoker	89 (55.6%)	21 (52.5%)	22 (55%)	23 (57.5%)	23 (57.5%)	0.931
Former smoker	44 (27.7%)	10 (25%)	13 (32.5%)	9 (22.5%)	12 (30%)	
Never smoker	27 (16.7%)	9 (22.5%)	5 (12.5%)	8 (20%)	5 (12.5%)	
Histological type						
Non-small cell lung cancer	118 (73.8%)	31 (77.5%)	30 (75%)	29 (72.5%)	28 (70%)	0.964
Small cell lung cancer	42 (26.2%)	9 (22.5%)	10 (25%)	11 (27.5%)	12 (30%)	
Comorbidities						
Hypertension	66 (41.2%)	14 (35%)	15 (37.5%)	17 (42.5%)	20 (50%)	0.432
Diabetes mellitus	53 (33.8%)	12 (30%)	14 (35%)	15 (37.5%)	15 (37.5%)	0.813
Chronic lung disease (COPD)	69 (43.1%)	15 (37.5%)	16 (40%)	19 (47.5%)	19 (47.5%)	0.798
BMI, mean ± SD	23.6 ± 3.3	24.2 ± 3.0	24.1 ± 3.2	23.8 ± 3.6	23.4 ± 3.7	0.337
Family history of cancer						
Yes	56 (35%)	13 (32.5%)	14 (35%)	12 (30%)	15 (37.5%)	0.974
No	104 (65%)	27 (67.5%)	26 (65%)	28 (70%)	25 (62.5%)	
ECOG performance status						
0-1	93 (58.1%)	31 (77.5%)	28 (70%)	21 (52.5%)	15 (37.5%)	0.001
≥2	67 (41.9%)	9 (22.5%)	12 (30%)	19 (47.5%)	25 (62.5%)	

Serum levels of PTH, CRP, lipid profile, and IL-6

PTH levels increased significantly from 10.8 ± 2.5 pg/mL in Stage 1 to 498 ± 89.4 pg/mL in Stage 4 (p < 0.001). Similarly, CRP levels rose from 5.8 ± 1.2 mg/L to 8.6 ± 2.4 mg/L (p = 0.004), indicating heightened systemic inflammation. While LDL levels showed minor fluctuations with no significant differences (p = 0.085), HDL levels declined significantly from 38 ± 5 mg/dL in Stage 1 to 31 ± 7 mg/dL in Stage 4 (p = 0.042), reflecting lipid profile deterioration. TAG levels progressively increased from 146 ± 15 mg/dL in Stage 1 to 165 ± 18 mg/dL in Stage 4 (p = 0.031). IL-6 levels exhibited a marked rise from 3.8 ± 0.9 pg/mL to 8.5 ± 2.1 pg/mL (p < 0.001), underscoring its role as a key inflammatory marker in lung cancer progression.

The 95% CIs provided insights into the precision and variability of these measurements. PTH CIs expanded from 10.00-11.60 pg/mL in Stage 1 to 469.41-526.59 pg/mL in Stage 4, while CRP CIs widened from 5.42-6.18 mg/L to 7.93-9.07 mg/L, emphasizing their progressive increase. LDL CIs remained stable, suggesting limited association with disease progression, whereas HDL CIs narrowed from 36.43-39.57 mg/dL to 29.14-32.86 mg/dL, reinforcing its relevance as a marker of metabolic disruption. TAG and IL-6 levels displayed increasing variability, with widening CIs reflecting their dynamic changes during disease progression.

These findings highlight significant differences in PTH, CRP, HDL, TAG, and IL-6 levels across stages (p < 0.05), while LDL remained unaffected. Additionally, a robust correlation was observed between BMI and ECOG performance status (r = 0.915, p < 0.001), linking higher BMI with better functional outcomes. These biomarkers offer critical insights for disease monitoring and personalized treatment strategies (Table 2).

**Table 2:** Mean, SD, and CI for serum levels of PTH, CRP, lipid profile, and IL-6 cross lung cancer stages One-way ANOVA was used to compare means across stages, with F values included, and a p value of <0.05 was considered statistically significant F values for the comparisons were estimated based on the corresponding p values. For PTH and IL-6, F values is found to be >10.0 (approximately 12.0-20.0). For CRP, the F value ranged between 6.0 and 8.0. For LDL, the F value was between 1.5 and 3.0, indicating a mild effect. HDL showed an F value in the range of 3.5-5.0, while TAG had an F value between 4.0 and 6.0 PTH: parathyroid hormone; CRP: C-reactive protein; LDL: low-density lipoproteins; HDL: high-density lipoproteins; TAG: triacylglyceride; IL-6: interleukin-6; SD: standard deviation; CI: confidence interval; ANOVA: analysis of variance

Parameter	Stage 1 (mean ± SD)	Stage 2 (mean ± SD)	Stage 3 (mean ± SD)	Stage 4 (mean ± SD)	95% CI				p value
					Stage 1	Stage 2	Stage 3	Stage 4	
PTH (pg/mL)	10.8 ± 2.5	12.4 ± 3.1	312 ± 65.2	498 ± 89.4	10.00-11.60	11.41-13.39	291.15-332.85	469.41-526.59	<0.001
CRP (mg/L)	5.8 ± 1.2	6.7 ± 1.5	8.4 ± 2.1	8.6 ± 2.4	5.42-6.18	6.22-7.18	7.73-9.07	7.83-9.37	0.004
LDL (mg/dL)	128 ± 10	140 ± 12	135 ± 15	140 ± 16	125.12-130.88	136.23-143.77	131.65-138.35	136.00-143.99	0.085
HDL (mg/dL)	38 ± 5	34 ± 4	30 ± 6	31 ± 7	36.43-39.57	32.74-35.26	28.14-31.86	29.14-32.86	0.042
TAG (mg/dL)	146 ± 15	155 ± 14	160 ± 16	165 ± 18	141.36-150.64	150.63-159.37	155.04-164.96	159.38-170.62	0.031
IL-6 (pg/mL)	3.8 ± 0.9	4.5 ± 1.2	7.2 ± 1.8	8.5 ± 2.1	3.51-4.09	4.11-4.89	6.72-7.68	7.93-9.07	<0.001

## Discussion

This study emphasizes the importance of biochemical and inflammatory markers in lung cancer progression. Elevated serum levels of PTH, CRP, and IL-6 with advancing disease stages highlight their potential as biomarkers for monitoring disease severity. This study shows elevated PTH levels consistent with known calcium metabolism and malignancy [13]. This rise can be due to tumor-induced metabolic changes and paraneoplastic syndromes that are typically seen with advanced cancer. These results highlight the need for further exploration of the prognostic utility of PTH and its role in cancer-associated metabolic complications.

CRP and IL-6, known markers of systemic inflammation, demonstrated significant increases with disease progression, consistent with their role in the tumor microenvironment. Elevated CRP levels likely reflect ongoing inflammatory responses, while IL-6, a proinflammatory cytokine, has been implicated in tumor growth, angiogenesis, and immune evasion [13,14]. These findings reinforce the importance of systemic inflammation in lung cancer and its clinical utility in disease monitoring and prognosis.

This study also highlights the systemic metabolic disruptions seen with lung cancer by focusing on alterations in lipid metabolism. Similarly, decreased HDL and elevated TAG levels support previous research regarding the metabolic dysregulation observed in cancer patients [15,16]. Oxidative stress and inflammation can elevate, decreasing HDL for its anti-inflammatory and antioxidant properties [17], and elevated TAG levels align with previous research highlighting metabolic dysregulation in cancer patients [15,16]. HDL, recognized for its anti-inflammatory and antioxidant properties, may decrease due to heightened oxidative stress and inflammation [17]. On the other hand, high TAG levels are a known feature of tumor-associated hyperlipidemia. Interestingly, although LDL levels were unchanged over disease stages, the other lipid profile changes highlight the need for complete metabolic assessment among lung cancer patients.

The variability observed in certain parameters, such as LDL levels, could be influenced by several factors, including demographic characteristics, comorbid conditions, and lifestyle habits like dietary intake and smoking status. These potential confounders may mask the direct impact of disease progression on lipid profiles, particularly LDL. Additionally, genetic predispositions and variations in systemic inflammatory responses could contribute to the observed patterns. Identifying and accounting for such confounders is essential to interpret the findings better. This interpretation provides insights into the multifactorial nature of biomarker variability and highlights the need for further research to delineate these influences.

The study also showed a decrease in ECOG performance status with an increase in disease stages, which can compromise functional capacity. It appears that better nutritional and metabolic reserves provide a positive correlation between BMI and ECOG performance status, such that individuals are more likely to have a better functional capacity even in more advanced disease stages. These findings are consistent with previously reported evidence that metabolic health may influence cancer outcomes [18,19]. The results of this study collectively emphasize the multifaceted changes involved in the metabolic and inflammatory-associated changes in lung cancer and their relevance to clinical monitoring and potential therapy.

Limitations

Due to the relatively small sample size (n = 160), the generalizability of the findings is limited. Furthermore, it was done at two institutions, which may not capture so much diversity. Conditions such as treatment regimens, comorbid conditions, and nutritional status, which might affect levels of biomarkers, were not controlled. Finally, with cross-sectional design, relationships of biomarkers with lung cancer progression may not be able to be presumed causal, and longitudinal studies are necessary for confirmation.

## Conclusions

The study revealed that PTH, CRP, IL-6, and lipid profile parameters play a significant role as markers of lung cancer progression. Levels of PTH, CRP, and IL6 increased progressively, whereas HDL levels declined and TAG levels increased, reflecting their roles in systemic inflammation and metabolic dysregulation. Overall, these findings highlight the importance of including these markers as part of routine clinical evaluations to detect disease severity and adjust treatment strategies. In addition, the strong correlation between ECOG performance status and BMI heightens the importance of functional and nutritional considerations in lung cancer treatment. Nonetheless, these results require further study in large scale and longitudinal studies to validate these findings, and to explore the possibility of prognostic and therapeutic integration into clinical practice.
